# Risk factors for brain metastases in patients with non–small‐cell lung cancer

**DOI:** 10.1002/cam4.1865

**Published:** 2018-11-08

**Authors:** Ning An, Wang Jing, Haoyi Wang, Ji Li, Yang Liu, Jinming Yu, Hui Zhu

**Affiliations:** ^1^ Department of Radiation Oncology Shandong Cancer Hospital and Institute, Shandong University Jinan China; ^2^ Department of Radiation Oncology Shandong Cancer Hospital and Institute, Shandong Academy of Medical Sciences Jinan China; ^3^ Department of Hematology Qilu Hospital, Shandong University Jinan China

**Keywords:** brain metastases, gene mutation, non–small‐cell lung cancer, prophylactic cranial irradiation

## Abstract

Brain metastases (BM) are severe incidents in patients with non–small‐cell lung cancer (NSCLC). The controversial value of prophylactic cranial irradiation (PCI) in NSCLC in terms of survival benefit prompted us to explore the possible risk factors for BM in NSCLC and identify the potential population most likely to benefit from PCI. Risk factors for brain metastases in NSCLC are reviewed in this article. Identifying patients with a higher risk of BM could possibly increase the benefit of PCI while reducing the discomfort and risks caused by unnecessary invasive procedures in the NSCLC patient population. Future studies might focus on finding a solid basis for the prediction of the occurrence of brain metastases and for the therapeutic decision on the use of PCI.

## Abstract 2

Brain metastases are a severe incident in patients with non–small‐cell lung cancer (NSCLC) and it is still common. Controversial value of prophylactic cranial irradiation (PCI) in NSCLC in terms of survival benefit prompted us to explore the possible risk factors for brain metastases (BM) in NSCLC and identify the potential population most likely to benefit from PCI. There are several risk factors of brain metastases in NSCLC. Identifying patients with higher risk of BM could possibly make them receive a greater benefit from PCI, then reduce the discomfort and the unnecessary risks caused by invasive procedures. Future study might take into consideration to find solid basis for therapeutic decisions.

## 1 INTRODUCTION

Non–small‐cell lung cancer (NSCLC) accounts for approximately 80%‐85% of all lung cancer cases, and 30%‐54% of NSCLC patients will develop brain metastases (BM) after treatment^1^, ^2^, ^3^, ^4^, ^5^; half of the patients with locally advanced NSCLC (LA‐NSCLC) may develop BM in the course of the disease.^6^, ^7^, ^8^ BM are the most common and severe complication of NSCLC because effective treatment is lacking. Patients with brain metastases, whose median survival time is <1 year, usually have a low quality of life and poor prognosis.^9^, ^10^, ^11^

Advances in multimodal therapeutic regimens have moderated the locoregional relapse of LA‐NSCLC and improved overall survival (OS).^6^, ^12^, ^13^ Systemic chemotherapy has led to improvements in the control of extracranial diseases and therefore in survival,^14^, ^15^ but its role in the management of brain metastases is limited because of the poor diffusion rate for drug molecules across the blood‐brain barrier (BBB).^14^, ^16^ Whole brain radiotherapy (WBRT) is widely used for the treatment of BM in NSCLC. Some studies have shown that radiosurgery or surgical resection combined with WBRT can effectively control brain metastases in LA‐NSCLC.^17^ However, in 2016, the ISRCTN3826061 study found that compared to the control group, BM patients exhibited little clinically significant benefit from WBRT.^18^

Prophylactic cranial irradiation (PCI) is the most effective method for preventing the morbidity associated with BM in NSCLC patients. Previous studies have shown that PCI was able to reduce the occurrence rate of BM by approximately 50%.^2^, ^19^, ^20^, ^21^, ^22^ However, many studies have shown that PCI might have no beneficial effect on OS.^19^, ^22^, ^23^, ^24^ In the RTOG‐0214 study, PCI failed to improve OS (*P *= 0.86; 1‐year OS 75.6% vs 76.9% for PCI vs observation, respectively).^23^ In 2012, the ASTRO 3014 study reported that although PCI could significantly reduce the incidence of BM, PCI had no effect on OS or might even be associated with a significant reduction in OS.^24^

These facts suggest that PCI may not be appropriate for all the patients with NSCLC. It is necessary to identify potential patients who may benefit from it. The evaluation of the risk factors for brain metastases to find the patients with NSCLC who may develop BM and benefit from PCI is meaningful. Although studies have focused on this problem,^25^, ^26^ a systematic summary is still necessary because of the rapid development of treatments for NSCLC, such as targeted therapy and immunotherapy. This review discusses several publications on the risk factors for BM and describes the risk factors for developing brain metastases in NSCLC patients.

## 2 PATHOLOGY

Non–small‐cell lung cancer can be further classified into squamous cell carcinoma, adenocarcinoma, and large cell carcinoma types, and many researchers in recent years have reported that patients with adenocarcinoma or non‐squamous cell carcinoma are more likely to develop BM.^4^, ^5^, ^25^, ^27^, ^28^, ^29^, ^30^

For operable NSCLC, the incidence of brain metastases in patients with adenocarcinoma was 2.86 times higher than that in non‐adenocarcinoma patients (95% CI: 1.58‐5.16, *P* = 0.001).^31^ Adenocarcinoma was also reported to be one of the predictive factors for BM (RR: 3.39; 95% CI: 1.78‐6.46 and *P* = 0.0002) in a group of stage I‐III NSCLC patients.^27^ There were also some studies comparing non‐squamous cell carcinoma with squamous cell carcinoma, which found that non‐squamous cell carcinoma was an independent risk factor for BM (*P* = 0.000; HR = 3.73; 95% CI: 2.25‐6.16).^25^ Therefore, non‐squamous cell carcinoma, especially adenocarcinoma, may be an independent risk factor for the metastasis of NSCLC.

However, there are different views. Some studies found no correlation between pathology and BM.^32^, ^33^, ^34^ As the study by Ceresoli reported, there was no statistically significant pathological effect when squamous cell carcinoma was compared with non‐squamous cell carcinoma.^32^ The possible explanation may be the variability caused by the method of brain metastases assessment. Jens et al reported in their study that brain autopsies were performed on 87 patients with adenocarcinoma, and 38 patients (44%) were BM‐positive.^35^ However, the frequency of BM in patients with adenocarcinoma who were clinically diagnosed was 24%‐35.7%.^36^, ^37^, ^38^

## 3 GENDER

The predictive value of gender for BM may still remain controversial. The results of several studies showed that the effects of gender on BM were limited, especially in LA‐NSCLC.^27^, ^39^ Earlier studies presented a meta‐analysis on risk factors for BM and found that gender could not be used as a marker to predict the development of BM.^26^ However, in patients with completely resected early‐stage NSCLC, being female may have predictive value for the incidence of BM.^30^ This conclusion was also confirmed in another study.^40^ Gender may have predictive value in early‐stage NSCLC, but this may not be suitable for LA‐NSCLC.

## 4 T AND N STATUSES AND TUMOR STAGES

Somearticles reported that the T and N statuses were both predictive factors. Bajard et al^27^ analyzed 305 patients with stage I‐III NSCLC and found that T4 (RR: 3.75; 95% CI: 1.72‐8.21; *P* = 0.0009) and N2‐3 (RR: 2.61; 95% CI: 1.32‐5.15; *P* = 0.0057) were risk factors for BM. A study of 264 patients with stage I‐IV NSCLC found that the incidence of BM increased with rising T and N stages (*P* = 0.05).^41^ More studies focused on the influence of N status on BM in NSCLC. Compared with the risk of BM in the absence of lymphatic involvement, the risk of brain metastases was significantly higher when the hilar (RR = 4.26 and *P* = 0.013) or homolateral mediastinal lymph nodes (RR = 5.49 and *P = *0.001) were metastatic.^36^ In the study by Ding et al,^30^ an increased risk of developing BM was associated with a number of metastatic lymph nodes >5 (*P = *0.000), LNR ≥30% (*P* = 0.000), number of metastatic mediastinal lymph nodes ≥3 (*P = *0.001), number of metastatic mediastinal lymph node stations between 2 and 4 (*P* = 0.000), and ratio of metastatic mediastinal lymph node stations >50% (*P* = 0.000). Regarding the relationship between tumor size or stage and BM, Robnett et al^29^ analyzed 150 patients with stage II‐III NSCLC and found that IIIB patients had an increased risk for BM (RR: 2.8; 95% CI: 1.3‐6.1). However, the relationship between tumor size or stage and BM was not found by Keith et al^34^ (stage IB‐IIIB) or Horinouchi et al^42^ (unresectable stage III). Another study did not find this relationship either.^25^ Most of the articles that we found showed that tumor stage has not previously been regarded as a predictive factor for brain metastases in resected NSCLC patients.^27^, ^43^, ^44^ Therefore, we concluded that lymph node metastases are an independent risk factor, especially when the mediastinal lymph nodes were multiple metastatic.

However, several studies failed to prove the T and N statuses were predictive factors.^10^, ^31^, ^34^, ^45^ The reasons underlying this difference need to be further explored. The possible reasons for this difference were due to only LA‐NSCLC being included in these studies and the variability in biology and pathology between unresectable and resectable NSCLC.

## 5 AGE

Many studies have reported that younger age is a risk factor for BM in NSCLC. They analyzed the predictive value of age and found that a patient aged younger than 60‐70 years of age was associated with a risk of brain metastases.^5^, ^28^, ^29^, ^32^, ^46^

Patient aged 60 years or younger was related to the incidence of BM (*P* = 0.004; HR = 2.03; 95% CI: 1.25‐3.32).^25^ In studies by Ceresoli et al,^32^ an age <60 years was a risk factor(OR = 1.26; 95% CI: 1.03‐1.53). Bajard et al^27^ considered an age <62 years to be a risk factor for brain metastases (RR = 2.5; 95% CI: 1.33‐4.76; *P* = 0.004). Dimitropoulos et al^33^ reported that the incidence rate of BM was reduced with increasing age (OR = 0.91; 95% CI: 0.87‐0.96; *P* < 0.001).

It was unclear why young patients have a higher BM risk. Several studies have shown that BM is associated with the angiogenic microenvironment, and the cerebrovascular microenvironment factors of young patients may be better than those of older patients.^47^ Whether there were differences in the expression of biomarkers, such as vascular endothelial growth factor, Ki‐67, and caspase‐3, between younger and older patients also needs further exploration.^48^ In addition, young people may have better performance statuses, which are associated with longer survival.

## 6 PERFORMANCE STATUS

The initial performance status following protocol entry was a predictor for brain metastases. Compared with patients in the group whose Karnofsky performance status was 50%‐60%, patients in the best performance groups (Karnofsky performance status >60%) developed metastases at a higher rate after protocol entry. In addition, the patients who responded to the treatment better may develop brain metastases during the therapy.^35^ However, neither difference was significant. In addition, in the study by Bajard, they did not find performance status to be a significant predictive factor.^27^

Patients in the best performance status groups have a somewhat higher risk for the development of brain metastases. Since the performance status has been shown to be a major prognostic factor for survival, differences in brain metastatic frequency may also be at least partially interpreted as the risk of such complications being changed at different times during disease progression.^35^

## 7 THE LEVELS OF TUMOR MARKERS

While most tumor markers are currently used in lung cancer examination, they may contribute to the clinical and histologic diagnosis of the disease, prognosis prediction, and therapeutic evaluation.^49^, ^50^

### 7.1 Neuron‐specific enolase (NSE)

As a key glycolytic enzyme, NSE is mainly expressed in neuroendocrine tumors but rarely expressed in NSCLC, except for in patients with neuroendocrine differentiation, which accounts for approximately 10% of NSCLC patients.^51^, ^52^, ^53^, ^54^

Recent studies of patients with locally advanced NSCLC have shown that NSE is an independent risk factor for BM.^55^ A multicenter retrospective study showed a high serum NSE level was an independent determinant of poor survival in patients with NSCLC with BM.^40^ NSE was identified as a significant predictive factor based on the stage I‐III NSCLC‐resected patients in the study by Zhang.^31^ In Ji's study, increased NSE and CA125 levels are both independent risk factors for BM.^25^ The association between elevated NSE levels and BM might reflect tumor heterogeneity, but the exact mechanism is unclear. No other reports referred to the relationship between CA125 levels and BM. Further clinical trials and functional studies are needed to validate these findings.

These results suggest that NSE plays a significant role in brain metastases, not only for locally advanced NSCLC but also for all stages of NSCLC.

### 7.2 Carcinoembryonic antigen (CEA)

Serum CEA levels are positively associated with advanced disease and tumor recurrence in resected NSCLC.^56^, ^57^, ^58^ Despite this well‐known association, there are few studies on the relationship between serum levels of CEA and brain metastases in advanced NSCLC.

Arrieta et al^39^ demonstrateda significant relationship between high CEA serum levels and BM development in adenocarcinoma compared with other histological types. In a prospective manner, they studied 293 patients with NSCLC in the IIIB‐IV clinical stage and found that CEA concentrations ≥40 ng/mL (RR 11.4; 95% CI, 1.7‐74; *P < *0.01) were an independent risk factor.^39^ This result is consistent with the study by Ma. That study revealed that CEA concentrations ≥23 ng/mL (*P = *0.001) were a predictive factor for developing BM after analyzing 134 patients with EGFR‐mutated advanced lung adenocarcinoma.^59^

The results above suggest that elevated serum CEA levels in NSCLC patients are an independent predictive factor for brain metastases.

## 8 ONCOGENE

Recently, targeted therapy that is based on the mutation state of molecular biomarkers has been developed for the treatment of non–small‐cell lung cancer.^60^ In fact, approximately 60%‐80% of the patients whose tumor samples contain somatic mutations in the kinase domain of the epidermal growth factor receptor (EGFR) gene respond to EGFR tyrosine kinase inhibitors (TKIs).^61^ Mutated KRAS, v‐erb‐b2 erythroblastic leukemia viral oncogene homolog 2 (HER‐2), and serine‐threonine kinase BRAF are also involved in the development and progression of NSCLC.^62^, ^63^

Although the molecular status of EGFR in primary NSCLC has been extensively studied, the data on the molecular status of BM from NSCLC are limited.^64^, ^65^, ^66^, ^67^, ^68^, ^69^ Studies of molecular pathways that mediate brain metastases have shown that oncogenes play an important role and that the molecular statuses of these genes need to be further investigated because they can be part of the patient risk stratification.^70^

### 8.1 EGFR mutations

It has been reported that the epidermal growth factor receptor (EGFR) mutation status could have an influence on the central nervous system (CNS) progression of NSCLC.^9^, ^39^, ^60^, ^69^, ^71^, ^72^ The study by Li et al analyzed 110 patients with NSCLC whose EGFR status was detected in the primary tumors and brain metastases. The EGFR mutation rates in patients with and without brain metastases were 64% and 31%, respectively, suggesting that brain metastases were more common in patients with EGFR mutations.^73^ However, this study had small sample sizes, which may have limitations. In 2016, a retrospective study including 1522 consecutive NSCLC patients reported that patients with EGFR mutations at the time of diagnosis have a nearly twofold higher risk of brain metastases.^74^ The results above suggest that the EGFR mutation status could have an influence on the CNS progression of NSCLC. The mechanisms for this phenomenon may be due to Met activation and epithelial‐mesenchymal transition (EMT).^74^, ^75^, ^76^ Some patients with EGFR mutations may undergo EMT, which may result in increased motility and invasiveness in their cancer cells.^76^

Heon et al found that the CNS progression of TKI‐treated NSCLC was dependent on the EGFR genotype. Compared with tumors with L858R mutations, tumors with exon 19 deletions showed a higher morbidity of CNS involvement (3% vs 21%, respectively).^77^ The patient's ethnic origin is also a determinant because the frequency of EGFR mutations is much higher in Asian patients (40%).^78^ Two studies found EGFR mutations in 44%‐63% of brain metastases in East Asian patients.^66^, ^71^ This is similar to the prevalence reported in primary tumors of East Asian people, which varies from 30% to 50%.^78^, ^79^ However, in the Caucasian population, only three studies reported the prevalence of EGFR mutations in BM, and they found that the rate was between 0% and 2%,^64^, ^65^, ^80^ which is far lower than the prevalence in primary tumors (10%).^81^, ^82^ In fact, the prevalence of EGFR mutations in NSCLC in non‐Asian patients is quite low in published studies.^80^

This difference maybe caused by patient variability because it is reported that EGFR mutations in NSCLC are significantly associated with the female gender and nonsmoking status.^77^, ^83^ Another hypothesis is that tumor heterogeneity at the molecular level may be responsible for the difference.

Another interesting point is that EGFR has been shown to be unstable during tumor progression. Italiano et al found that discordant EGFR expression can reach 33.3% in a cohort of 30 matched primary tumors and metastases.^84^

In conclusion, EGFR mutations play an important role in the metastasis of NSCLC and may support risk stratification, especially in East Asian patients, but the status of these mutations needs to be further investigated.

### 8.2 ALK mutations

The overall incidence rate of BM in patients withanaplastic lymphoma kinase (ALK)‐positive NSCLC is high.^85^, ^86^, ^87^ Some studies have shown that patients with ALK overexpression mutations have stable or asymptomatic BM at the initial diagnosis or with progression. In the study of 21 patients with NSCLC with mutated ALK by Deepa Rangachari,^88^ 23.8% (5/21) of patients had BM at the initial diagnosis. Over time, the cumulative incidence of BM increased after diagnosis: 23.8% at 1 year, 45.5% at 2 years, and 58.4% at 3 years. Although the clinical data were not enough to build a convincing conclusion, we can still find that the ALK mutations are associated with the incidence of BM in NSCLC.

### 8.3 KRAS mutations

In contrast to EGFR, there are relatively few reports concerning the V‐Ki‐ras2 Kirsten rat sarcoma viral oncogene homolog (KRAS) status in the brain metastases of lung cancer patients.^64^, ^68^, ^69^, ^89^

In the study by Villalva, KRAS mutational status did not facilitate the metastasis of NSCLC.^60^ This finding is consistent with several studies that showed that KRAS mutations were related to the suppression of progression.^90^, ^91^ The overall mutation rate of KRAS in primary tumors was not significantly different from that of matched metastases in a meta‐analysis by Wang; the odds ratio was 1.224 (95% CI: 0.808‐1.856, *P* = 0.340).^92^ That study did not find significant differences in overall KRAS mutation rates between primary and metastatic NSCLC either.

### 8.4 PD‐1/PD‐L1 and TMB

Immune evasion has been seen as an important feature of the tumor. The expression of PD‐1/PD‐L1 and tumor mutational burden (TMB) is the main predictive factors of the state of the tumor. The number of studies about the relationships between the expression of PD‐1/PD‐L1 or TMB and brain metastases is relatively small.

Some studies supported the conclusion that TMB is associated with BM. TMB was significantly higher in NSCLC with BM than in NSCLC with other types of metastases.^93^ The current evidence shows that TMB is different in NSCLC with BM compared to other types of NSCLC.^93^ Further study is required to identify the relationship between TMB and BM.

To our knowledge,there are few reports studying the relationship between the expression of PD‐1/PD‐L1 and BM. Several studies reported higher PD‐L1 expression in primary tumor compared with matched brain metastases (52% vs 32%, respectively).^94^ Few studies have reported that the expression of PD‐1/PD‐L1 is a predictive factor of BM.

## 9 CONCLUSIONS

Patients with locally advanced NSCLC are at high risk for CNS relapse. Previous review articles reported that non‐squamous cell carcinoma type, elevated serum CEA level, and lymph node metastases (especially multiple metastases in the mediastinal lymph nodes) are independent risk factors for brain metastases^25^, ^26^ (Table S1). We collected more articles, summarized the latest studies and found that adenocarcinoma type, younger age (<60 years), and elevated serum NSE levels also need to be considered (Table S1).

With the development of targeted therapy and gene testing, the predictive value of oncogenes should be better assessed. EGFR mutations have been reported to be an independent risk factor for brain metastases. Although there was not sufficient evidence, ALK mutation and high TMB were also believed to be associated with high BM incidence (Table S1).

According to several nomograms for predicting brain metastases that were published in recent years, patients with an increased number of risk factors had a much higher risk of BM.^31^, ^43^ Non‐squamous cell carcinoma type, especially adenocarcinoma; lymph node metastases; and high levels of serum tumor markers largely predict the occurrence of brain metastases.^31^, ^43^ However, these nomograms did not cover the effects of oncogenes, which may play an increasingly valuable role in the future.

Detection of a higher risk of BM could possibly identify patients who would receive a greater benefit from PCI and thus reduce the discomfort and risks associated with unnecessary invasive procedures. Therefore, the stratification of patients with newly diagnosed NSCLC using risk factors for brain metastases could have important meaning. There have been published nomograms that attempt to build models that include the risk factors for brain metastases to provide risk estimates, but a more solid basis for therapeutic decisions needs to be created in the future.

## CONFLICT OF INTEREST

None declared.
