# Supplementary material for: Risk factors for brain metastases in patients with non–small‐cell lung cancer
Source: Cancer Med. 2018 Nov 8;7(12):6357–64. doi: 10.1002/cam4.1865 (PMC6308070; doi:10.1002/cam4.1865)
Supplement: Supplementary file 1 [file CAM4-7-6357-s001.docx]

Table 1. Main Characteristics of the Studies Included on Risk Factors for BM^a^

| Study | Num. | Stage | T-status | N-status | Histology | Gender | Age | PS | CEA level | NSE level | EGFR+ | ALK+ |
| --- | --- | --- | --- | --- | --- | --- | --- | --- | --- | --- | --- | --- |
| Jacot  et al. (2001) | 231 | I- IV |  |  |  | HR:2.29  (1.26,4.16) *p*=0.006 | <63  HR:1.2  (1.003,1.043) *p*=0.021 |  |  | > 12.5 ng/ml  HR:1.72  (1.11,2.68) *p*=0.016 |  |  |
| Ceresoli et al. (2002) | 112 | IIB-IIIA |  | OR:1.18 (0.96,1.45) | OR:1.07*  (0.76,1.15) |  | <60  OR:1.26  (1.03,1.53) |  |  |  |  |  |
| Bajard  et al.  (2004) | 305 | I-II/III | RR:3.75（1.72,8.21）*p*=0.0009 | RR: 2.61  (1.32,5.15)*p*=0.0057 | RR:3.39**  (1.78,6.46)*p*=0.0002 |  | <62  RR:2.5  (1.33, 4.76)  *p*=0.004 | RR:0.36  (0.12,1.12)  *p*=0.078 |  |  |  |  |
| Arrieta  et al.  (2009) | 293 | IIIB-IV |  |  | *p*=0.0002** | *p*=0.86 | <60  *p*=0.79 |  | >20 ng/ml  *p* <0.001 |  | *p*=0.2 |  |
| Wang  et al.  (2009) | 223 | III | *p*=0.936 | *P<0.001* | *p*=0.029* |  |  |  |  |  |  |  |
| Petrović et al.  (2011) | 107 | IIIA | *p*=0.652 | *P<*0.001 | *p*=0.021* | *p*=0.652 | <60  *p*=0.535 |  |  |  |  |  |
| Horinou-chi et al.  (2012) | 116 | III |  |  |  |  |  |  | >20 ng/ml  HR:2.64  (1.39,5.02)  *p*= 0.01 |  |  |  |
| Ding et al.  (2012) | 217 | IIIA | *p*=0.596 | *p*=0.001 | RR:4.13*  (1.86,9.19) *p*=0.001 | *p*=0.012 | <60  *p*=0.035 |  |  |  |  |  |
| Ji et al.  (2014) | 346 | III |  |  | HR:3.726*  (2.25,6.16)  *p*=0.000 |  | <60  HR:0.491 (0.301,0.801) *p*=0.004 |  |  | >18 ng/ml  HR:1.968  (1.196,3.238)  *p*=0.008 |  |  |
| Shaw et al.  (2014) | 130 |  |  |  |  |  |  |  |  |  |  | Presence of brain metastases:49% |
| Shin et al.  (2016) | 286 | I- IV |  |  |  |  |  |  |  |  | OR:9.68  (2.42,40.45) *p*=0.002 |  |
| Zhang  et al.  (2016) | 637 | I- III | HR:1.24  (0.54–2.81) *p*=0.337 | HR:0.57  (0.18,1.79) *p*=0.615 | HR:2.86*  (1.58,5.16) *p*=0.001 | HR:1.57  (0.85,2.88) *p*=0.149 |  |  | >5 ng/ml  HR:1.19  (0.70,2.04) *p*= 0.519 | >60 ng/ml  HR:9.52  (3.48,26.04) *p<*0.001 |  |  |
| Bhatt  et al.  (2017) | 1522 | I- IV |  |  |  |  |  |  |  |  | OR: 1.8 *p<*0.001 |  |

a. Table included every independent risk factors and listed the studies that supported it. While analyzing risk factors for BM, OR, HR, RR, 95% CIs and p value were used to estimate the effect of each study. For each study, the data was described depending on the results provided in a previous publication, which directly reported in the original study.

*squamous cell carcinoma vs. non-squamous cell carcinoma; **adenocarcinoma vs. non-adenocarcinoma; HR, hazard ratio; OR, odds ratio; RR, relative risk; CEA, carcinoembryonic antigen; NSE, Neuron-specific Enolase; PS, Eastern Cooperative Oncology Group performance status
